# Carbon emissions versus value-added in export-driven countries: case of Vietnam

**DOI:** 10.1186/s40008-022-00272-w

**Published:** 2022-09-08

**Authors:** Phuong Thao Nguyen

**Affiliations:** 1 Graduate School of Global Governance, Meiji University, Tokyo, Japan; 2 National Center for Socio-Economic Information and Forecast, Ministry of Planning and Investment Vietnam, Hanoi, Vietnam

**Keywords:** Exports, Value-added, Carbon emissions, Economics, Environment, Vietnam

## Abstract

Manufacturing for export is gradually becoming the main pillar of economic growth in many developing countries, including Vietnam. Since 1986, Vietnam has adopted an open economic policy and promoted trade activities. Therefore, Vietnam’s exports have significantly increased and contributed to economic development. The benefits of exports are undeniable, but Vietnam also faces serious environmental problems caused by these activities. This paper analyzes the impact of Vietnam’s export activities on economics and the environment through comparison between carbon emissions and value-added embodied in exports using an input–output model, then provides some recommendations to adjust Vietnam's export strategy in the future. The main findings indicate that carbon dioxide emissions (CO_2_) embodied in exports have increased from 2006 to 2015. The carbon intensity of exports increases, while the value-added intensity decreases. As compared with production for the domestic market, production for domestic demand creates faster value-added and slower carbon emissions than production for exports. This study suggests that Vietnam should reform its export structure alongside technological improvements and other policy adjustments to curb Vietnam’s growing CO_2_ emissions.

## Introduction

Vietnam’s export strategy is an important part of its development strategy. After "Doi moi" in 1986, Vietnam's exports have increased continuously, especially, after joining the World Trade Organization (WTO) in 2007, trade activities have been increasingly promoted. Vietnam identified exports as the priority activity. Exports were considered to play an important role in creating a breakthrough for economic growth. Since then, Vietnam has achieved a high growth rate, with an annual average growth rate of 6.2% between 2005 and 2020. In addition, exports have become a major driver of economic growth (Eckhardt et al., 2018; Chaponnière and Cling, 2009), and expanding production for exports has contributed to an increase in employment for Vietnamese (Lim, 2011). Exports are expanding and contributing to Vietnam’s growth but exports of the foreign direct investment (FDI) sector account for the main proportion. In 2005, the share of FDI’s exports was 57%. The figure in 2020 rose to 72.3%, which shows the strong growth of the FDI's exports and the dependence of Vietnam’s exports on the FDI.

Although it is undeniably Vietnam export achievements, the export efficiency, and its negative impacts on the environment are becoming the top concerns of Vietnam's government. The country still participates at a low stage of the global value chain and only performs steps that bring the lowest value-added such as assembly and processing. In addition, the low localization rate in export products is also a problem. Moreover, carbon emissions have been increasing because of industrial development, which causes uncontrollable consequences. Total carbon emissions in Vietnam have increased continuously, especially in big cities and industrial zones. According to the latest national GHG inventory of 2013 published under the 2nd Biannual Update Report (BUR) of Viet Nam in 2017, the energy and industrial processes sectors are key factors that account for 80% of total carbon emissions in Vietnam. According United Nations Framework Convention on Climate Change (UNFCCC) 2019, fossil fuel accounts for 58% in total global carbon emissions. However, currently, fossil fuels (coal, oil, and gas) are the dominant source for energy production in Vietnam. This is the reason for energy production, and especially electricity production, is a major factor within emissions in Vietnam. Tran (2019) shows that electricity consumption and generation in Vietnam increased significantly from 1990 to 2016; however, fuel for electricity generation comes mainly from hydro and fossil fuels: hydro (38.9%); coal (32.6%), and gas (27.2%) while renewables and oil only accounted for 0.2% and 0.7%, respectively.

In the context of the complicated situation from the COVID_19 pandemic and the trade conflicts among China and other countries, investment flows are shifting to Asia countries, especially ASEAN countries (ASEAN Secretariat, 2021). Their trade is expected to play an increasingly important role in global trade in the next periods. Exports in Vietnam are also forecasted to increase significantly, but the concerns are whether export activities would lead to the thriving of carbon emissions in the country. However, the previous studies only focus on developed and highest emissions countries. In addition, previous studies focused either on carbon emission or value-added in trade, but the studies did not consider these two factors systematically. Therefore, the policy-makers could not have overview of costs and benefits that exports bring to the countries, which lead to the limitation in adjusting policies. This research aims to consider the benefits and costs of exports through evaluating carbon emissions and value-added embodied in exports in Vietnam. In addition, this study also compares between production for exports and the domestic market. Thereby, it could enlighten domestic policy-makers on adjusting investment attraction policies, restructuring economic activities towards sustainable development. This study also could contribute in literature an example of examining carbon emissions in exports to guiding and adjusting policies of emerging countries, and it could be a lesson for others with similar conditions. The remainder of the paper is structured as follows. Section “Literature review” provides a literature review on this topic. The next section introduces the method and data. Section 4 shows results and discussions. Section 5 summarizes our findings.

## Literature review

Research on the environmental and economic impacts of trade has received ample attention in the literature recently. A lot of studies consider the environmental impact through assessing CO_2_ embodied in exports and the economic impact through the value-added embodied in exports by using the input–output model, especially studies for China, it is due to the country is a key in the global supply chain and also the world's largest emitter.

In term of carbon emissions content in exports, there are a large number of studies on emissions in trade in China. Liu et al. (2010) examined CO_2_ emissions embodied in bilateral trade between Japan and China showed that the carbon content in China's export to Japan decreased from 1995–2000 while Jayanthakumaran and Liu (2016) indicated that the more energy efficient a country among trading partners was, the lower the overall global CO_2_ emissions there will be when considering CO_2_ emissions content in bilateral trade between Australia and China. Analyzing carbon emissions content in trade for top trading partners of China, Bin (2013) showed that transitions of China’s emissions embodied in imports to those in the exports account for around 4.6–13.3% of total emissions. Huang et al. (2019) examined embodied CO_2_ emissions in China’s exports to its top 20 trade partners based on the emissions embodied in bilateral trade (EEBT) approach. They estimated that more than 20% of total production-based CO_2_ emissions are exported to other countries. While CO_2_ emissions exported to developed countries decreased significantly, CO_2_ emissions exported to developing countries increased sharply. However, Fan et al. (2019) show that higher trade openness leads to the reduction of both CO_2_ emissions intensity and gross CO_2_ emissions in the industrial sectors. In addition, there is some literature on carbon emissions embodied in India's exports. Zhu (2018) found that carbon emissions embodied in India's exports are from mainly the iron and steel industry and the service industry. The carbon emissions embodied in India's exports accounted for one-fifth of India’s total emissions from 2013 to 2014. Banerjee (2019), by adding over all the sectors, assessed the CO_2_ emissions embodied in Indian exports increases from 163.3 Mt in 1995 to 398.5 Mt in 2009. Therefore, carbon emissions more than doubled within the 14 years from 1995 to 2009. Banerjee also found that the scale effect is the main reason for the increase in emissions. Recently, Kim and Tromp (2021) investigated carbon emissions embodied in trade in South Korea between 2000 and 2014 by employing MRIO and found significant increases in emissions embodied in exports and imports, which lead to an increase in total emissions in the country. Wu et al. (2022) calculated onshoring and offshoring emissions of 43 countries from 2000 to 2014 by using the Emissions Embodied in Bilateral Trade (EEBT) approach, and found that the main reason for offshoring emissions in the USA and onshoring emissions in China is the trade balance, which is not related to the trade composition. In contrast, Germany and Japan's onshoring emissions are mainly driven by trade composition. Japan is likely to export brown products and to import green products.

In term of economic impacts of exports, value-added content in exports are considered. Chen (2004) introduced a framework using the input–output model to estimate the increases in domestic value-added (or equivalently, GDP) when its exports increase. They found that Chinese exports to the world will induce an increase in China's GDP more than those of Chinese exports to the US. They showed that the domestic value-added of processing exports is lower than those of the non-processing exports. In considering both value-added and CO_2_ emissions in China’s exports, Dietzenbacher et al. (2012), by making a distinction between the production of processing exports, production for domestic use only, and other production, found an overestimation of emissions in China's exports. In addition, processing exports are relatively clean compared to other kinds of production, but the processing exports also generate less value-added compared to non-processing exports. Liu et al., (2015) examined value-based emissions and carbon emissions content in the value-added chain. This study indicated the emissions embodied in the value-added chains of China increased rapidly, but the net emissions embodied in value-added chain transfers were small and decreasing.

Although a bunch of research on carbon emissions embodied in exports of largest emission countries and some developed countries in the world, the research in other economies with a rapid increase of carbon emissions is lacking. In addition, previous studies focused either on carbon emission or value-added in trade, but the studies did not consider these two factors systematically. Therefore, the policy-makers could not have an overview of the costs and benefits that exports bring to the countries, which leads to the limitation in adjusting policies. In the next period, the investment flows tend to shift to Asia countries, especially ASEAN countries. The research on carbon emissions and value-added in trade, especially in exports should be more focused on these countries, thereby guiding these countries in orienting to attract investment and restructuring exports toward sustainable development.

In Vietnam, Bui et al. (2011) used the input–output model to analyze the effect of export on import and value-added in Vietnam, the results show that exports are one of reason causing trade deficit in Vietnam while exports induced to value-added lower than other factors of final demand. Nguyen et al. (2019) analyzed the included effect of final demand including exports on GHG using an input–output model. They showed that exports are one key factor for carbon emissions in Vietnam. Nguyen et al. (2018) used input–output structural decomposition analysis to show the main drivers of carbon dioxide emissions in Vietnam. They found that exports were a key driving force incremental changes in CO_2_ emissions during the periods 2000–2007 and 2007–2011. Changes in exports caused a sharp increase in carbon emissions from the first period (23,908 kt) to the second period (46,665 kt). However, in these studies, the amount of carbon emissions in Vietnam's exports was not determined. They also have not indicated which industries/countries are the main cause of emissions, and thus these studies have not been able to provide clear policy recommendations for Vietnam.

To fill the gap, this study will apply input–output analysis to examine the embodied CO_2_ emissions and embodied value-added in Vietnam’s exports to its largest trading countries, and then provide suggestions for adjusting Vietnam's export policies. This study also could contribute in literature an example of examining carbon emissions and value-added in exports in the export-driven country, raising the attention in costs and benefits from export-oriented production in these countries.

## Methodology and data

### Methodology

Input–output theory was introduced by Wassily Leontief in 1941. In his study, the first input–output tables were constructed for the US in 1919, 1929 and 1941 (Leontief, 1941). The input–output table is considered as the basis of the System of National Accounts (SNA) of the United Nations (United Nations, 1953). According to Wassily Leontief, “input–output analysis describes and explains the level of input of each sector of a given national economy in terms of its relationships to the corresponding levels of activities in all the other sectors” (Leontief, 1970). In other words, the input–output table describes the overall picture of the economy, the relationship between consumption and production, and the interaction between sectors. Therefore, the input–output model is applied in economic analysis and forecasting and is also expended to suit environmental analysis.

Carbon emissions and value-added content in exports are mainly calculated by employing emissions embodied in bilateral trade (EEBT) and multi-region input–output (MRIO) approaches. While the MRIO approach has advantages in analyzing final consumption, the EEBT is better in analyzing trade and climate policy (Peters, 2008). At the national level, policy-makers are more likely to be interested in emissions embodied in domestic demand and in exports (Peters and Hertwich (2008), Peters and Solli (2010), Huang et al (2019)). Therefore, this study will apply the EEBT approach to calculate carbon emissions embodied in exports. In addition, an expansion of the EEBT approach will be applied to calculate the value-added embodied in exports. Table 1 shows the format of the input–output table used in the EEBT model. In this input–output table, exports are classified by country.

**Table 1:**
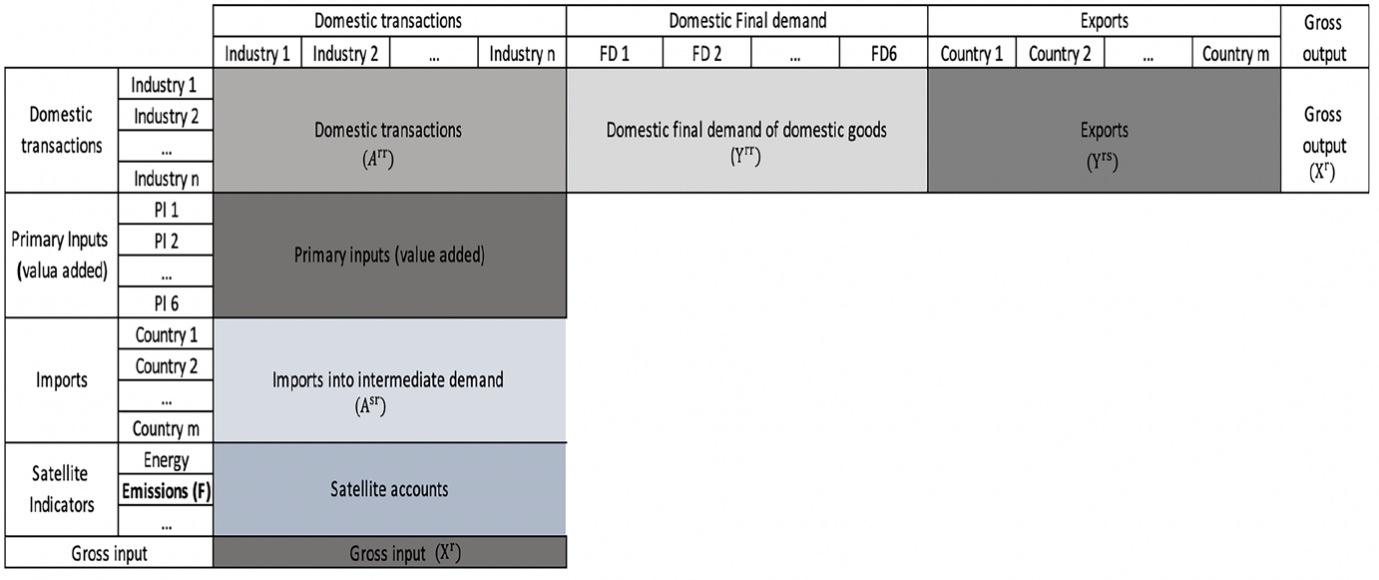
Format of input–output table in the EEBT approach

Source: Own construction based on Eora multi-region input–output database

The multi-region input–output relationship is:

1 $$\left(\begin{array}{c}{X}_{1}^{r}\\ \vdots \\ {X}_{n}^{r}\end{array}\right)= \left(\begin{array}{ccc}{A}_{11}^{rr}& \cdots & {A}_{1n}^{rr}\\ \vdots & \ddots & \vdots \\ {A}_{n1}^{rr}& \cdots & {A}_{nn}^{rr}\end{array}\right)\left(\begin{array}{c}{X}_{1}^{r}\\ \vdots \\ {X}_{n}^{r}\end{array}\right)+ \left(\begin{array}{c}{\sum Y}_{1}^{rr}\\ \vdots \\ {\sum Y}_{n}^{rr}\end{array}\right)+\left(\begin{array}{c}{\sum Y}_{1}^{rs}\\ \vdots \\ {\sum Y}_{n}^{rs}\end{array}\right),$$

where *r* and *s* denote different regions, in this research, *r* is the region being studied and *s* denotes the exported partners of *r*; ${X}_{i}^{r}$ is total output of region *r* of sector *i*; ${A}^{rr}$ is an input–output coefficient matrix for region *r*, its elements ${A}_{ij}^{rr}$ represents the amount of good *i* required as an intermediate input for the production of one unit of good *j* in region *r,* and *i, j* = *1…n,* ${\sum Y}_{i}^{rr}$ *is* total row of ${Y}_{i}^{rr}$ which is the domestic final consumption in region *r* of sector *i;*${Y}^{rs}$ is an export matrix from region *r* to region *s* ($1\le s\le \mathrm{m}$, and $s\ne r)$ and ${Y}_{i}^{rs}$ is a vector corresponding to the *i*th row of${Y}^{rs}$*.*

#### CO_2_ emissions embodied in domestic demand and in exports

The carbon emissions generated by the domestic demand of region *r* or carbon emission embodied in the domestic demand (EED) can be obtained from:

2 $$\left(\begin{array}{c}{EED}_{1}^{rr}\\ \vdots \\ {EED}_{n}^{rr}\end{array}\right)=\left(\begin{array}{ccc}{f}_{1}^{rr}& \dots & 0\\ \vdots & \ddots & \vdots \\ 0& \cdots & {f}_{n}^{rr}\end{array}\right) {\left(\begin{array}{ccc}I-{A}_{11}^{rr}& \dots & -{A}_{1n}^{rr}\\ \vdots & \ddots & \vdots \\ -{A}_{n1}^{rr}& \dots & I-{A}_{nn}^{rr}\end{array}\right)}^{-1}\left(\begin{array}{c}{\sum Y}_{1}^{rr}\\ \vdots \\ {\sum Y}_{n}^{rr}\end{array}\right),$$

and the carbon emissions embodied in exports (EEE) from region *r* to regions:

3 $$\left(\begin{array}{c}{EEE}_{1}^{rs}\\ \vdots \\ {EEE}_{n}^{rs}\end{array}\right)=\left(\begin{array}{ccc}{f}_{1}^{rr}& \dots & 0\\ \vdots & \ddots & \vdots \\ 0& \cdots & {f}_{n}^{rr}\end{array}\right) {\left(\begin{array}{ccc}I-{A}_{11}^{rr}& \dots & -{A}_{1n}^{rr}\\ \vdots & \ddots & \vdots \\ -{A}_{n1}^{rr}& \dots & I-{A}_{nn}^{rr}\end{array}\right)}^{-1}\left(\begin{array}{c}{Y}_{1}^{rs}\\ \vdots \\ {Y}_{n}^{rs}\end{array}\right),$$

where $1\le s\le m$, and $s\ne r$; $${f}^{rr}= \left(\begin{array}{ccc}{f}_{1}^{rr}& \dots & 0\\ \vdots & \ddots & \vdots \\ 0& \cdots & {f}_{n}^{rr}\end{array}\right)$$ is the diagonal matrix of sectoral energy/emissions coefficient of region *r,* its elements of the main diagonal can be calculated as:

4 $${f}_{i}^{rr}=\frac{{F}_{i}^{rr}}{{X}_{i}^{r}},$$

with ${F}_{i}^{r}$ as carbon emission of sector *i* in region *r*.

It should be noted that this research only considers CO_2_ emissions, and thus the matrix ${f}^{rr}$ in above equations is region-specific CO_2_ emissions per unit of industry output.

Then, total CO_2_ emissions embodied in the exports of region *r* are:

5 $${EEE}^{r}={\sum }_{s\ne r}\sum_{i=1}^{n}{EEE}_{i}^{rs},$$

with ${EEE}_{i}^{rs}$ as elements of vector $$\left(\begin{array}{c}{EEE}_{1}^{rs}\\ \vdots \\ {EEE}_{n}^{rs}\end{array}\right)$$, and represents CO_2_ emissions embodied in exports from region *r* to region *s* of sector *i*.

Total CO_2_ emissions embodied in the domestic demand of region *r* are:

6 $${EED}^{r}={\sum }_{i=1}^{n}{EED}_{i}^{rr},$$

where ${EED}_{i}^{rr}$ is an element of vector $$\left(\begin{array}{c}{EED}_{1}^{rr}\\ \vdots \\ {EED}_{n}^{rr}\end{array}\right)$$, representing CO_2_ emissions embodied in the domestic demand of sector *i* in region *r*.

Total production-based CO_2_ emissions in region *r* are:

7 $${F}^{r}= {EED}^{r}+{EEE}^{r}.$$

Equation (7) shows that total emissions in region *r* are not only caused by domestic demand of region r, but also caused by foreign demand represented by exports.

#### Value-added embodied in domestic demand and in exports

Similarly, value-added embodied in domestic demand and in exports can be calculated as:

8 $$\left(\begin{array}{c}{VED}_{1}^{rr}\\ \vdots \\ {VED}_{n}^{rr}\end{array}\right)=\left(\begin{array}{ccc}{v}_{1}^{rr}& \dots & 0\\ \vdots & \ddots & \vdots \\ 0& \cdots & {v}_{n}^{rr}\end{array}\right) {\left(\begin{array}{ccc}I-{A}_{11}^{rr}& \dots & -{A}_{1n}^{rr}\\ \vdots & \ddots & \vdots \\ -{A}_{n1}^{rr}& \dots & I-{A}_{nn}^{rr}\end{array}\right)}^{-1}\left(\begin{array}{c}{\sum Y}_{1}^{rr}\\ \vdots \\ {\sum Y}_{n}^{rr}\end{array}\right),$$

and

9 $$\left(\begin{array}{c}{VEE}_{1}^{rs}\\ \vdots \\ {VEE}_{n}^{rs}\end{array}\right)=\left(\begin{array}{ccc}{v}_{1}^{rr}& \dots & 0\\ \vdots & \ddots & \vdots \\ 0& \cdots & {v}_{n}^{rr}\end{array}\right) {\left(\begin{array}{ccc}I-{A}_{11}^{rr}& \dots & -{A}_{1n}^{rr}\\ \vdots & \ddots & \vdots \\ -{A}_{n1}^{rr}& \dots & I-{A}_{nn}^{rr}\end{array}\right)}^{-1}\left(\begin{array}{c}{Y}_{1}^{rs}\\ \vdots \\ {Y}_{n}^{rs}\end{array}\right),$$

where $1\le s\le m$, and $s\ne r$; $${v}^{r}=\left(\begin{array}{ccc}{v}_{1}^{rr}& \dots & 0\\ \vdots & \ddots & \vdots \\ 0& \cdots & {v}_{n}^{rr}\end{array}\right)$$ is a diagonal matrix with direct value-added coefficients along the diagonal that are determined by:

10 $${v}_{i}^{rr}=\frac{{V}_{i}^{rr}}{{X}_{i}^{r}},$$

where ${V}_{i}^{rr}$ is total value-added of sector *i* in region *r,* ${X}_{i}^{r}$ is gross input of sector *i* in region *r.*

Total value-added embodied in the exports of region *r* is

11 $${VEE}^{r}={\sum }_{s\ne r}{\sum }_{i}{VEE}_{i}^{rs},$$

where ${VEE}_{i}^{rs}$ is an element of vector $$\left(\begin{array}{c}{VEE}_{1}^{rs}\\ \vdots \\ {VEE}_{n}^{rs}\end{array}\right)$$, representing value-added embodied in the exports of sector *i* from region *r* to region *s.*

Total value-added embodied in the domestic demand of region *r* is:

12 $${VED}^{r}={\sum }_{i=1}^{n}{VED}_{i}^{rr},$$

where ${VED}_{i}^{rr}$ is an element of vector $$\left(\begin{array}{c}{VED}_{1}^{rr}\\ \vdots \\ {VED}_{n}^{rr}\end{array}\right)$$, representing value-added embodied in the domestic demand of sector *i* in region *r.*

Total value-added in region *r* is

13 $${V}^{r}= {VED}^{r}+{VEE}^{r}.$$

Equation (13) shows that total value-added in region r is not only caused by domestic demand of region *r*, but also caused by foreign demand represented by exports.

#### Carbon dioxide emissions intensity of exports and value-added intensity of exports

Carbon dioxide emissions intensity of exports of region *r* to region *s* measures CO_2_ emissions embodied in one unit of exports of region *r* to region *s* (${CIE}^{rs}$), and can be calculated as follows:

14 $${CIE}^{rs}=\frac{{\sum }_{i}{EEE}_{i}^{rs}}{\sum_{i}{E}_{i}^{rs}}.$$

Similarly, value-added intensity of exports of region *r* to region *s* measures value-added embodied in one unit of exports of region *r* to region *s (*$${VIE}^{rs})$$, and can be calculated as follows:

15 $${VIE}^{rs}=\frac{{\sum }_{i}{VEE}_{i}^{rs}}{\sum_{i}{E}_{i}^{rs}}.$$

### Data

In Vietnam, there is a lack of environmental data, especially environmental data by sector. In addition, the different sector classifications among economic and environmental accounts also cause difficulties in collecting data. Therefore, this study uses Vietnam’s input–output tables taken from the Eora multi-region input–output (MRIO) database, which is the only source providing information about input–output tables matching with environmental indicators for Vietnam though this database provides estimated data for Vietnam. In addition, in this database, the greenhouse gas satellite accounts include carbon emissions data from three sources including Emission Database for Global Atmospheric Research (EDGAR), Carbon Dioxide Information Analysis Center (CDIAC), and the PRIMAP (EORA, 2015). In this paper, carbon emissions from EDGAR are used due to the total carbon emissions from this source being closer to total carbon emissions from the national GHG inventories of Vietnam in the Biennial Update Report (BUR) of Viet Nam (MONRE, 2017).

This database also provides a time series of input–output tables with environmental and social indicators for 190 countries (Lenzen et al., 2012, 2013). The format of the table is presented in Table 1. For this study, the time series of Vietnam’s input–output tables from 2006 to 2015 (the latest table is from 2015) is used to analyze carbon emissions embodied in Vietnam’s exports. These tables contain primary input and final demand blocks, imports and exports classified by partner (190 countries), and environmental indicators for 113 sectors. To simplify analyses, total domestic final demands are summed up from household final consumption, non-profit institutions serving households, government final consumption, gross fixed capital formation, changes in inventories, and acquisitions less disposals of valuables. For exports, this study considers the top 15 trading partners for Vietnam's exports, including Japan, China, Australia, South Korea, USA, Germany, France, Hong Kong, Indonesia, Malaysia, Netherlands, Singapore, Taiwan, Thailand, and the UK. Other export destinations are aggregated into ROW (rest of the world). In addition, in this paper, the aggregation of 113 sectors into 18 sectors is performed after the calculation to avoid the environmentally sensitive sectors being aggregated with other sectors in the aggregated sector classification and causing bigger errors during the calculation process (Appendix 1).

## Empirical results

### Carbon dioxide emissions and value-added embodied in Vietnam’s exports

Vietnam's production-based CO_2_ emissions^1^ have increased continuously from 82 million tons (Mt) CO_2_ in 2006 to 175.2 Mt CO_2_ in 2015, with an average increase of 11.4% per year (see Table 2). This has been caused by growth in both carbon emissions embodied in domestic demand and exports. However, the expansion of carbon emissions embodied in each component is different. Total carbon emissions embodied in Vietnam's exports experienced a significant increase from 22.2 Mt CO_2_ in 2006 to 57.5 Mt CO_2_ in 2015, with an average annual growth rate of 15.8%. In addition, Table 2 shows that around 27 to 36% of total Vietnam’s production-based CO_2_ emissions were caused by foreign demand for each year during the period 2006 to 2015. The proportion of emissions owning to foreign demand followed a rising trend from 27% in 2006 to 32% in 2015, peaking at 36% in 2014. For carbon emissions embodied in domestic demand, total carbon emissions embodied in domestic demand accounts for around 60% of total carbon emissions, but the share of carbon emissions embodied in the domestic demand has decreased. This is due to the annual growth rate of carbon emissions embodied in exports (15.8%) being higher than in the domestic demand (9.7%).

**Table 2:** CO_2_ emissions embodied in exports and domestic demand in 2006–2015

	Total (Mt)	Domestic demand		Exports	
		CO_2_ (Mt)	as % of total	CO_2_ (Mt)	As % of total
2006	81.95	59.71	72.86	22.24	27.14
2007	92.65	67.43	72.77	25.22	27.23
2008	106.43	78.17	73.45	28.26	26.55
2009	121.43	91.01	74.95	30.42	25.05
2010	134.26	98.99	73.73	35.27	26.27
2011	140.92	102.73	72.90	38.19	27.10
2012	142.78	104.50	73.19	38.28	26.81
2013	146.47	109.89	75.03	36.58	24.97
2014	158.42	100.61	63.51	57.81	36.49
2015	175.15	117.68	67.19	57.47	32.81

In term of value-added embodied in exports, value-added embodied in exports also increased from 2006 to 2015 with an average annual growth rate of 8.7% (see Table 3); however, this is much lower than the growth of carbon emissions embodied in exports. From a different perspective of domestic demand, value-added content in domestic demand accounts for the main proportion of total value-added. The growth rate of value-added content in domestic demand (10.2%) is higher than value-added content in exports growth (8.7%).

**Table 3:** Value-added embodied in domestic demand and exports in 2006–2015

	Total (billion USD)	Domestic demand		Exports	
		Value-added (billion USD)	as % of total	Value-added (billion USD)	as % of total
2006	53.57	37.02	69.11	16.55	30.89
2007	59.05	39.84	67.47	19.21	32.53
2008	71.61	50.62	70.68	21.00	29.32
2009	68.19	50.11	73.49	18.08	26.51
2010	57.87	42.57	73.55	15.30	26.45
2011	62.96	45.87	72.86	17.09	27.14
2012	71.35	52.61	73.73	18.74	26.27
2013	79.13	59.79	75.56	19.34	24.44
2014	109.67	73.50	67.02	36.17	32.98
2015	105.57	74.62	70.68	30.96	29.32

**Fig. 1:**
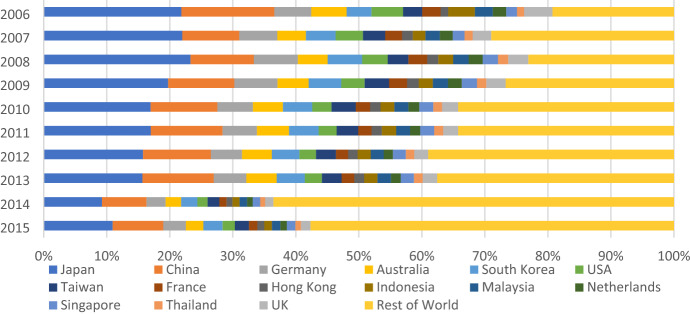
The share of Vietnam’s CO_2_ emissions embodied in exports to different countries in 2006–2015

By country, from 2006 to 2013, exported CO_2_ emissions that were caused by the 15 top export partners accounted for 60 to 80% of total carbon emissions embodied in exports. However, this proportion decreased significantly in 2014–2015 to only 30–40% (see Fig. 1). This can be explained due to the share of export turnover to these countries decreasing (see Appendix 2). With increasing participation in free trade agreements and the trade promotion efforts of Vietnam's government, the expansion and diversification of export markets has caused changes in the share of export turnover by market. In addition, after recovering from the global economic crisis, exporters are well aware of the benefits of diversifying export markets and have been searching for new export markets.

Japan, China, Germany, South Korea, and Australia are the top five CO_2_ importers of Vietnam. In 2015, CO_2_ emissions embodied in exports to Japan, China, and Germany, respectively, occupied 10.9%, 8.0%, and 3.6% of the country’s total exported embodied CO_2_ emissions. These countries were followed by South Korea (3.0%) and Australia (2.8%). For the two largest carbon importers of Vietnam, while China's carbon emissions imports from Vietnam increased from 2.2 Mt CO_2_ to 4.6 Mt CO_2_, the carbon emissions import of Japan did not change too much (around 5.8 Mt CO_2_) due to the export turnover from Vietnam to China increasing much higher than exports to Japan from 2006 to 2015. This becomes clearer when considering carbon intensity by sector, as is discussed in the next section.

**Fig. 2:**
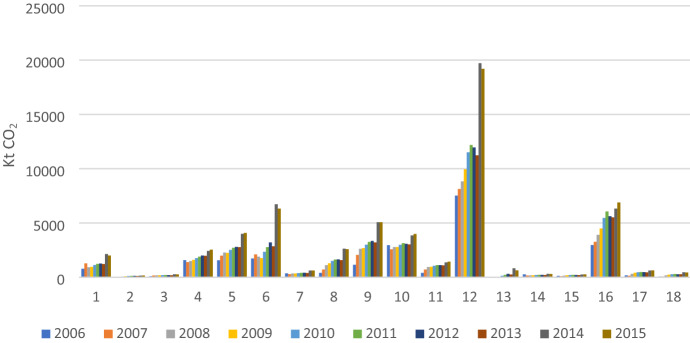
Carbon emissions embodied in Vietnam’s exports by sector in 2006–2015

By sector, Fig. 2 shows that the bulk of CO_2_ is emitted by only five industries including sector 12 (Electricity, Gas, Water), sector 16 (Transportation), sector 6 (Building materials); sector 9 (Machinery and Equipment), and sector 10 (Textiles and Wearing Apparel). This result reflects the relatively strong dependence of production for export on the energy and transportation sectors. These sectors are essential intermediate inputs in the production of exports. In addition, Sector 12 (Electricity, Gas, and Water) plays a dominant role in Vietnam's embodied CO_2_ in exports, which is not surprising, given the fact that fossil fuels such as hydro and coal still dominate electricity production in Vietnam.

In addition, manufacturing sectors such as sector 6 (Building materials); sector 9 (Machinery and Equipment); and sector 10 (Textiles and Wearing Apparel), which also cause significant amounts of CO_2_ emissions embodied in Vietnam’s exports, are energy-intensive sectors. In other words, the energy and energy-intensive sectors are the main contributors to Vietnam's export emissions. This situation is similar to China where focus is on production based on cheap labor and energy—a low level of outsourcing (Huang et al., 2019; Dietzenbacher et al., 2012).

### Carbon dioxide emission intensity and value-added intensity of exports

Carbon intensity of exports measures CO_2_ emissions embodied in one unit of exports. Table 4 shows the carbon intensity of Vietnam’s exports, which increased from 0.99 kg CO_2_ per 1 USD of exports in the period 2006–2010 to 1.14 kg CO_2_ per 1 USD of exports in 2011–2015. This means that Vietnam’ export products have become more carbon intensive. On the other hand, the value-added intensity of exports decreased from 0.63 USD per 1 USD of exports in the period 2006–2010 to 0.59 USD per 1 USD of exports in 2011–2015. The results clearly show that Vietnam is increasingly exporting products with low value-added content.

**Table 4:** Carbon intensity and value-added intensity of exports

	2006–2010	2011–2015
Value-added intensity of exports	0.6297	0.5976
Carbon emissions intensity of exports	0.9955	1.1415

**Fig. 3:**
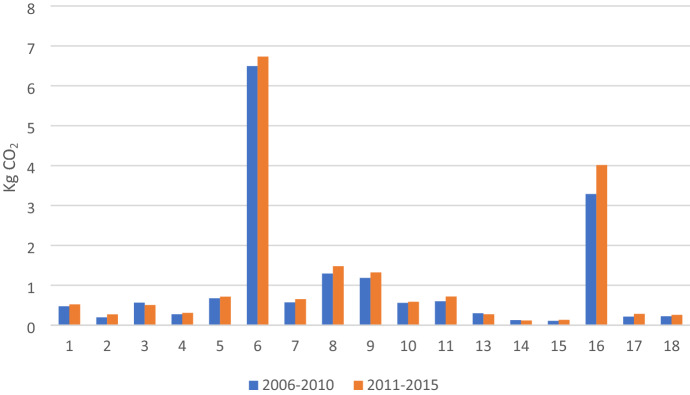
Carbon emissions intensity of Vietnam’s exports by sector

By sector, sector 12 (Electricity, Gas, Water) has exceptional carbon intensity compared with other sectors, which is a characteristic of an energy industry in Vietnam that mainly depends on fossil fuels (see Appendix 3). Currently, around half of Vietnam’s electricity generation comes from coal while coal is the dominant CO_2_ emissions source related to electricity generation (Johnson et al., 2021). After excluding the outlier (sector 12), the top two CO_2_ emissions intensive industries are sector 6 (Building materials) and sector 16 (transportation), which can explain the high total carbon emissions embodied in exports of these sectors (see Fig. 3).

In addition, Fig. 3 also shows that almost all manufacturing sectors such as sector 8 (Chemical and Non-metallic products), sector 9 (Machinery and Equipment), sector 10 (Textiles and Wearing Apparel) and sector 11 (Other manufacturing) have high carbon intensity. In contrast, agricultural sectors, including sector 1 (Agriculture), sector 2 (Forestry), sector 3 (Fishery), and service sectors, including sector 14 (Trade and repair), sector 15 (Hotel and Restaurant), sector 17 (Financial Intermediation and Business Activities), sector 18 (Other services) have low carbon intensity.

**Fig. 4:**
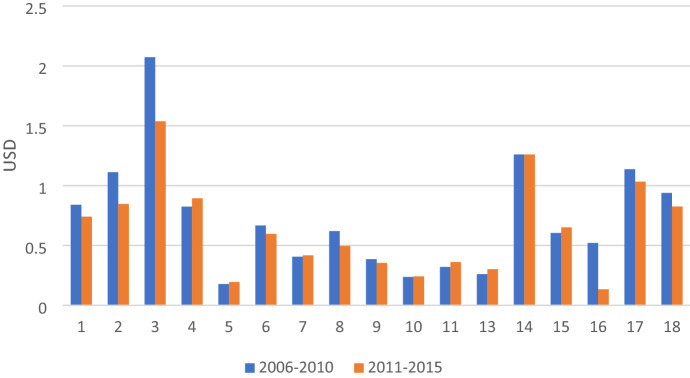
Value-added intensity of Vietnam’s exports by sector

In terms of value-added intensity by sector, the top CO_2_ emitting industries such as sectors 6, 16, 8, 9, 10, and 11 play a more modest role in generating value-added, whereas sectors 1, 2, 3, 14, 15, 17, and 18 create more value-added (see Fig. 4).

**Fig. 5:**
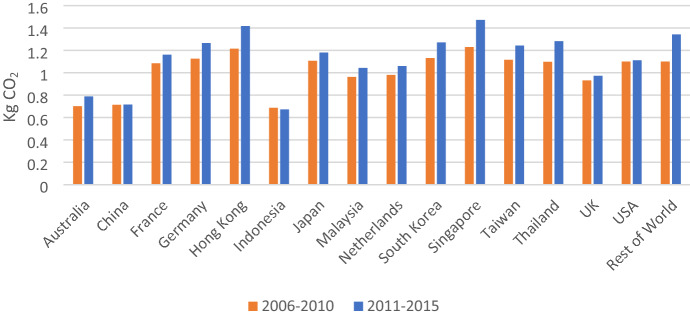
Carbon intensity of Vietnam’s exports by country

By country, Indonesia, China, and Australia are countries receiving Vietnam's exports with the smallest carbon intensity. The carbon intensity of Vietnam's export to Indonesia, China and Australia are, respectively, 0.67, 0.72, and 0.78 kg CO_2_ per 1 USD of exports. In contrast, the carbon intensity of Vietnam's exports to Singapore, Hong Kong and Germany are the highest at 1.47, 1.42, and 1.27 KgCO_2_ per 1 USD, respectively. Moreover, while the carbon intensity of exports to almost countries has increased, the figure for Indonesia decreased and for China was almost unchanged (see Fig. 5).

**Fig. 6:**
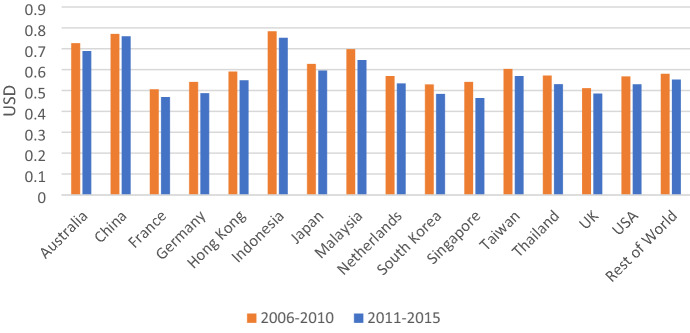
Value-added intensity of Vietnam’s exports by country

In term of the value-added intensity of exports by country, exports to Indonesia, China, and Australia are more value-added intensive with a value-added intensity of export, respectively, 0.75, 0.76, and 0.68 USD per 1 USD of exports. In contrast, Vietnam’s exports to Hong Kong and Germany are lower value-added intensive with a value-added intensity of exports at 0.55 and 0.49 USD per 1 USD of exports, respectively (see Fig. 6).

The differences in carbon intensity and value-added intensity of Vietnam’s exports by country are explained by comparing the export structure of Vietnam to these countries. For example, China and Indonesia import more agricultural products and Australia imports service sectors from Vietnam whereas Germany, Hong Kong, and Singapore import more manufacturing products such as sector 9 (Machinery and Equipment), and sector 10 (Textiles and Wearing Apparel).

## Conclusion and policy implications

### Discussion and policy implications

The results above show that the export activities are creating increasing emissions while their value-added has a slower growth rate. More importantly, the carbon intensity of exports is increasing while the value-added intensity is decreasing. It means that Vietnam's export production is coming to the bottom of the smiling curve of the global value chain. In other words, the production for export has not been as effective as Vietnam expected. Vietnam issued a series of preferential policies for export production, and these activities are expected to become the main drivers of Vietnam's growth. However, it seems that exports only play a role in growth relying on energy, natural resources, and cheap labor and not sustainable growth. In addition, it should be noted that the majority of Vietnam's exports come from the FDI sector. Selecting FDI for promoting exports should be considered carefully toward enhancing the value-added and mitigating emissions.

When exports activities are ineffective and the domestic market is neglected, Vietnam's economy will face risks. The domestic market will be dominated by international wholesalers and retailers. It should be known that the Vietnamese market is a potential market with a population of 95.54 million (in 2018); moreover, the income of Vietnamese people has risen from low to middle income. Therefore, domestic demand is increasing. If the government continues to give priority to exports, businesses producing goods for the domestic market will be at a disadvantage. In addition, exports depending heavily on the FDI sector also put Vietnam at risk. This is because with the fluctuations of the international market, FDI to Vietnam can be redirected to other countries and cause a crisis for Vietnam's economy. Moreover, it may cause the abuse of foreign investors in obtaining investment licenses and preferential policies.

Notably, the results of analyzing carbon embodied in exports by sectors have demonstrated that most of the sectors that cause high emissions are energy and energy-intensive industries. As discussed in introduction part, the energy industry in Vietnam relies on fossil energy and therefore emission levels are very high. However, due to lax environmental regulations, which are quite common in developing countries, Vietnam still attracts investment with medium and low production technologies that produce high levels of emissions in the production process. If export production does not apply modern, eco-friendly, and low-energy consuming technologies, these activities will continue to generate high levels of emissions in Vietnam. Most developing countries attract FDI with the expectation of technology transfer. However, according to a survey of the Vietnam Ministry of Planning and Investment (MPI, 2013), most of the technology that the FDI sector has shifted to Vietnam is mid-range and high energy-consuming technologies. The application of these technologies to export production areas is greatly affecting the environment in Vietnam.

In addition, due to the strategy of industrialization development, there is a lack of attention paid to the agricultural sectors. Agricultural land area is decreasing due to the conversion of agricultural land to the industrial zones. However, this study has shown that the agricultural sectors create high value-added and low emissions. Vietnam appears to be over-focusing on the export turnover of manufacturing industries and neglecting the agricultural sector. In addition to the agriculture sector, the service sector is also a potential sector with high value-added intensity and low carbon emission intensity of exports. However, given the limitation of labor quality and investment, the scale of this sector is still smaller compared to other sectors.

### Concluding remarks

Vietnam has grown from being a poor country. The country’s economic opening, especially regarding export activity, has contributed to its high growth rate achievement. Undoubtedly, exports bring benefits to Vietnam. However, this study has also shown that exports are increasingly causing pollution in Vietnam. Carbon content in exports is increasing, and Vietnam is increasingly exporting carbon-intensive products, while the exports of value-added intensive products are decreasing. Therefore, stricter environmental policies need to be promulgated and implemented for all enterprises in Vietnam in general, but also in particular for enterprises with export activities. It is time for export activities to be reconsidered in terms of sustainable economic growth. In addition, FDI's exports now account for a major share of total exports and mainly export manufacturing products. Therefore, in particular, Vietnam needs to consider carefully in selecting FDI flows, and encouraging FDI with eco-friendly technology processes, and low energy consumption to improve technology in processing export production. Currently, due to the US–China conflict and the Covid-19 pandemic, the trend of diversifying supply chains and shifting investment out of China is increasingly clear and countries diversify their sources of supply, which gives Vietnam more opportunities to attract FDI. Therefore, Vietnam should take advantages to select cleaner FDI for sustainable development. In general, Vietnam needs to consider promoting sustainable investment such as ESG (Environmental, Social, Governance) investment in production, especially in export production.

At the sectoral level, the energy and energy-intensive sectors are still the main driver of carbon emissions whereas the agriculture and services sectors emit lower levels of carbon emissions and generate more value-added. Therefore, Vietnam should consider restructuring and encouraging agricultural products and the service sector. Besides, technology improvement and renewable energy development also need to be considered to reduce the carbon intensity of energy and energy-intensive sectors. Finally, as compared between the production for exports and the production for domestic demand through carbon emissions embodied and value-added embodied in exports and the domestic demand, the study also shows that production for domestic demand has created faster value-added and slower carbon emissions than production for exports. Moreover, with a population of 95.54 million (in 2018) and the increase in income per capita, the domestic market is a potential market that Vietnam needs to exploit instead of over-focusing on export markets.

## Data Availability

The datasets used and/or analyzed during the current study are available from the corresponding author on reasonable request.

## Appendix 1 Sector aggregation

**Table:**

No.	Aggregated sectors	Original sectors
1	Agriculture	Paddy (all kinds)
		Raw rubber
		Coffee beans
		Sugarcane
		Tea
		Other crops
		Pig (all kinds)
		Cow (all kinds)
		Poultry
		Other livestock
		Irrigation service
		Other agricultural services
2	Forestry	Forestry
3	Fishery	Fishery
		Fish–farming
4	Mining and quarrying	Coal
		Metallic ore
		Stone
		Sand, gravel
		Other non-metallic minerals
		Crude oil, natural gas (except exploration)
5	Food, beverages and tobacco	Processed, preserved meat and by products)
		Processed vegetables, and animals’ oils and fats
		Milk, butter and other dairy products
		Cakes, jams, candy, coca, chocolate products
		Processed and preserved fruits and vegetables
		Alcohol, beer and liquors
		Beer and liquors
		Non-alcohol water and soft drinks
		Sugar, refined
		Coffee, processed
		Tea, processed
		Cigarettes and other tobacco products
		Processed seafood and by products
		Rice, processed
		Other food manufactures
6	Building materials	Glass and glass products
		Ceramic and by products
		Bricks, tiles
		Cement
		Concrete, mortar and other cement products
		Other building materials
7	Wood, paper and recreation products	Paper pulp and paper products and by products
		Processed wood and wood products
8	Chemical and non-metallic products	Basic organic chemicals
		Basic inorganic chemicals
		Chemical fertilizer
		Fertilizer
		Pesticides
		Veterinary
		Health medicine
		Processed rubber and by products
		Soap, detergents
		Perfumes and other toilet preparation
		Plastic (including semi-plastic products)
		Other plastic products
		Paint inl, varnish and other painting materials
		Other chemical products
9	Machinery and equipment	Health instrument and apparatus
		Precise and optics equipment, meter (all kinds)
		Home appliances and its spare parts
		Motor vehicles, motorbikes and spare parts
		Bicycles and spare parts
		General-purpose machinery
		Other general-purpose machinery
		Other special-purpose machinery
		Automobiles
		Other transport mean
		Electrical machinery
		Other electrical machinery and equipment
		Machinery used for broadcasting, television and information activities
		Non-ferrous metals and products (except machinery equipment)
		Ferrous metals and products (except machinery equipment)
10	Textiles and wearing apparel	Weaving of cloths (all kinds)
		Fibers, thread (all kinds)
		Ready-made clothes, sheets (all kinds)
		Carpets
		Weaving and embroidery of textile-based goods (except carpets)
		Products of leather tanneries
		Leather goods
		Animal feeds
11	Other manufacturing	Products of printing activities
		Products of publishing house
		Other physical goods
12	Electricity, gas, water	Gasoline, lubricants (already refined)
		Electricity, gas
		Water
13	Construction	Civil construction
		Other construction
14	Trade and repair	Trade
		Repair of small transport means, motorbikes and personal household appliances
15	Hotel and restaurant	Hotels
		Restaurants
16	Transportation	Transportation
		Railway transport services
		Water transport services
		Air transport services
17	Financial intermediation and business activities	Banking, credit, treasury
		Lottery
		Insurance
		Science and technology
		Real estate
		Real estate business and consultancy services
18	Other services	Communication services
		Tourism
		State management, defense and compulsory social security
		Education and training
		Health care, social relief
		Culture and sport
		Association
		Other services
		Reexport/reimport

## Footnotes

1 Production-based CO_2_ emissions = CO_2_ emissions embodied in domestic demand + CO_2_ emissions embodied in exports.
